# Sex differences in in-hospital management in patients with sepsis and septic shock: a prospective multicenter observational study

**DOI:** 10.1038/s41598-024-55421-x

**Published:** 2024-02-28

**Authors:** Sejoong Ahn, Bo-Yeong Jin, Sukyo Lee, Sungjin Kim, Sungwoo Moon, Hanjin Cho, Kap Su Han, You Hwan Jo, Kyuseok Kim, Jonghwan Shin, Gil Joon Suh, Woon Yong Kwon, Tae Gun Shin, Han Sung Choi, Sangchun Choi, Yoo Seok Park, Sung Phil Chung, Won Young Kim, Hong Joon Ahn, Tae Ho Lim, Sung-Hyuk Choi, Jong-Hak Park, Korean Shock Society (KoSS) Investigators, Sang-Min Kim, Seung Mok Ryoo, Gun Tak Lee, Sung Yeon Hwang, Byuk Sung Ko, Sung-Joon Park, Jin Ho Beom, Taegyun Kim, Yoon Sun Jung, Juhyun Song, Taeyoung Kong, Eunah Han, Ji Eun Hwang, Hui Jai Lee, Gu Hyun Kang, Kihwan Choi, Ki Young Jeong, Seok Hun Ko, Hyo Jin Bang, Jinwoo Jeoung, Min Joon Seo, Sangsoo Han, Heewon Yang, Chiwon Ahn, Changsun Kim, Hyungoo Shin

**Affiliations:** 1 Department of Emergency Medicine, Korea University Ansan Hospital, 123, Jeokgeum-ro, Danwon-gu, Ansan-si, 15355 Gyeonggi-do Republic of Korea; 2 Department of Biomedical Sciences, Seoul National University College of Medicine, Seoul, Republic of Korea; 3 Department of Emergency Medicine, Korea University Anam Hospital, Seoul, Republic of Korea; 4 Department of Emergency Medicine, Seoul National University College of Medicine, Seoul National University Bundang Hospital, Seongnam, Gyeonggi-do Republic of Korea; 5 Department of Emergency Medicine, CHA Bundang Medical Center, CHA University, Seongnam, Republic of Korea; 6 Department of Emergency Medicine, Seoul Metropolitan Government-Seoul National University Boramae Medical Center, Seoul, Republic of Korea; 7 Department of Emergency Medicine, Seoul National University Hospital, Seoul, Republic of Korea; 8 Department of Emergency Medicine, Samsung Medical Center, Sungkyunkwan University School of Medicine, Seoul, Republic of Korea; 9 Department of Emergency Medicine, College of Medicine, Kyung Hee University, Seoul, Republic of Korea; 10 Department of Emergency Medicine, Soonchunhyang University Bucheon Hospital, Bucheon, Republic of Korea; 11 Department of Emergency Medicine, Yonsei University College of Medicine, Seoul, Republic of Korea; 12 Department of Emergency Medicine, Gangnam Severance Hospital, Yonsei University College of Medicine, Seoul, Republic of Korea; 13 Department of Emergency Medicine, Asan Medical Center, University of Ulsan College of Medicine, Seoul, Republic of Korea; 14 Department of Emergency Medicine, Chungnam National University Hospital, Daejeon, Republic of Korea; 15 Department of Emergency Medicine, College of Medicine, Hanyang University, Seoul, Republic of Korea; 16 Department of Emergency Medicine, Korea University Guro Hospital, 148, Gurodong-ro, Guro-gu, Seoul, 08308 Republic of Korea; 17 Asan Medical Center, Seoul, South Korea; 18 Samsung Medical Center, Seoul, South Korea; 19 Hanyang University Seoul Hospital, Seoul, South Korea; 20 Korea University Guro Hospital, Seoul, South Korea; 21 Severance Hospital, Seoul, South Korea; 22 Seoul National University Hospital, Seoul, South Korea; 23 Gangnam Severance Hospital, Seoul, South Korea; 24 Seoul National University Bundang Hospital, Seoul, South Korea; 25 Seoul Metropolitan Government-Seoul National University Boramae Medical Center, Seoul, South Korea; 26 Hallym University Kangnam Sacred Heart Hospital, Seoul, South Korea; 27 CHA Gumi Medical Center, Gumi-si, South Korea; 28 Kyung Hee University Hospital, Gangdong, South Korea; 29 Seoul St. Mary’s Hospital, College of Medicine, Seoul, South Korea; 30 Dong-A University Hospital, Busan, South Korea; 31 University Bucheon Hospital, Bucheon-si, South Korea; 32 Ajou University Hospital, Suwon-si, South Korea; 33 Chung-Ang University Hospital, Seoul, South Korea; 34 Hanyang University Guri Hospital, Guri-si, South Korea

**Keywords:** Disparities, Antibiotics, Fluid, Sepsis, Septic shock, Emergency department, Infectious diseases, Fever, Risk factors, Medical research

## Abstract

Sex differences in the in-hospital management of sepsis exist. Previous studies either included patients with sepsis that was defined using previous definitions of sepsis or evaluated the 3-h bundle therapy. Therefore, this study sought to assess sex differences in 1-h bundle therapy and in-hospital management among patients with sepsis and septic shock, defined according to the Sepsis-3 definitions. This observational study used data from Korean Shock Society (KoSS) registry, a prospective multicenter sepsis registry. Adult patients with sepsis between June 2018 and December 2021 were included in this study. The primary outcome was adherence to 1-h bundle therapy. Propensity score matching (PSM) and multivariable logistic regression analyses were performed. Among 3264 patients with sepsis, 3129 were analyzed. PSM yielded 2380 matched patients (1190 men and 1190 women). After PSM, 1-h bundle therapy was performed less frequently in women than in men (13.0% vs. 19.2%; *p* < 0.001). Among the bundle therapy components, broad-spectrum antibiotics were administered less frequently in women than in men (25.4% vs. 31.6%, *p* < 0.001), whereas adequate fluid resuscitation was performed more frequently in women than in men (96.8% vs. 95.0%, *p* = 0.029). In multivariable logistic regression analysis, 1-h bundle therapy was performed less frequently in women than in men [adjusted odds ratio (aOR) 1.559; 95% confidence interval (CI) 1.245–1.951; *p* < 0.001] after adjustment. Among the bundle therapy components, broad-spectrum antibiotics were administered less frequently to women than men (aOR 1.339, 95% CI 1.118–1.605; *p* = 0.002), whereas adequate fluid resuscitation was performed more frequently for women than for men (aOR 0.629, 95% CI 0.413–0.959; *p* = 0.031). Invasive arterial blood pressure monitoring was performed less frequently in women than in men. Resuscitation fluid, vasopressor, steroid, central-line insertion, ICU admission, length of stay in the emergency department, mechanical ventilator use, and renal replacement therapy use were comparable for both the sexes. Among patients with sepsis and septic shock, 1-h bundle therapy was performed less frequently in women than in men. Continuous efforts are required to increase adherence to the 1-h bundle therapy and to decrease sex differences in the in-hospital management of patients with sepsis and septic shock.

## Introduction

Sepsis is a life-threatening organ dysfunction caused by a dysregulated host response to infection^1^. The increasing incidence and mortality rates associated with sepsis have resulted in a global health burden^2,3^. Sepsis is a medical emergency and, therefore, resuscitation and management should be initiated promptly^4^. Bundle therapy plays a key role in the early resuscitation of patients with sepsis. The recently introduced 1-h bundle therapy^5^ includes the following key components: lactate measurement, obtaining blood cultures before antibiotic administration, broad-spectrum antibiotic administration, adequate fluid resuscitation, and vasopressor application, for patients in whom vasopressor support is indicated. Besides bundle therapy, various in-hospital managements significantly affect patient survival in sepsis.

Despite standardized international guidelines, there exist sex differences in the in-hospital management of sepsis^6–8^ and other critical diseases^9–11^ that may lead to sex-specific differences in survival outcomes. To ensure improved survival by providing better resuscitation and management for patients of both sexes with sepsis and septic shock, sex differences in the 1-h bundle therapy and in-hospital management must be evaluated in accordance with current guidelines and the improvement of resuscitation and management should be based on sex difference of 1-h bundle therapy and in-hospital management. Despite the establishment of the Sepsis-3 definition and introduction of the 1-h bundle therapy, the majority of studies on sex differences in bundle therapy or in-hospital management either included patients with sepsis that was defined according to previous definitions^6,8,12,13^ or evaluated the 3-h bundle therapy^7,12^.

Therefore, this study aimed to assess sex differences in 1-h bundle therapy and in-hospital management among patients with sepsis and septic shock, which were defined in accordance with the Sepsis-3 definitions. We hypothesized that 1-h bundle therapy and in-hospital management would be performed less frequently in women than in men.

## Methods

### Study design and setting

This observational study used data from the Korean Shock Society (KoSS) registry—a prospective multicenter sepsis and septic shock registry that has assimilated data from 15 university teaching hospitals in the Republic of Korea since 2015^14,15^. Adult patients with suspected or confirmed infection, and patients with either hypotension despite fluid resuscitation, or requiring vasopressor support, or hyperlactemia were enrolled in the KoSS registry. Patients with a do-not-resuscitate order, with a diagnosis of sepsis at ≥ 6 h after admission to the emergency department, who were transferred from other hospitals without the inclusion criteria of the KoSS upon arrival at the emergency department, and who were transferred to another hospital from the emergency department were excluded from the KoSS registry. The data collection for the KoSS registry was approved by the Institutional Review Board of each participating hospital.

This study was conducted in accordance with the principles of the Declaration of Helsinki. The Institutional Review Board of the Korea University Ansan Hospital approved this study (2023AS0109) and waived the requirement for informed consent because of the observational nature of the study.

### Study population

Adult patients who were diagnosed with sepsis according to the Sepsis-3 definition^1^ and enrolled in the KoSS registry between June 2018^5^ and December 2021 were included in this study. Patients with unknown time variables for the 1-h bundle component and those with missing variables for calculating the Sequential Organ Failure Assessment (SOFA) and Acute Physiologic Assessment and Chronic Health Evaluation II (APACHE II) scores were excluded from the study.

### Definitions and data collection

Sepsis was defined as an acute increase in the SOFA score ≥ 2 from the baseline that was caused by infection^1^. Septic shock was defined as a serum lactate level ≥ 2 mmol/L and the requirement of a vasopressor despite adequate fluid resuscitation to maintain a mean arterial pressure (MAP) ≥ 65 mmHg^1^. Sepsis patients were managed according to the Surviving Sepsis Campaign guidelines^16^. The 1-h bundle component comprised lactate measurement, obtaining blood cultures before antibiotic administration, broad-spectrum antibiotic administration, rapid fluid resuscitation of 30 mL/kg for patients with hypotension or lactate ≥ 4 mmol/L, and vasopressor administration for patients with hypotension despite fluid resuscitation to maintain MAP ≥ 65 mmHg. Time zero was defined as the triage time in the emergency department^5^.

The following data were extracted from the KoSS registry: age, sex, comorbidities, infection focus, initial vital signs, laboratory results, SOFA, and APACHE II scores, in-hospital management including 1-h bundle therapy, and survival outcomes.

### Outcomes

The primary outcome was adherence to the 1-h bundle therapy, in accordance with the Surviving Sepsis Campaign of 2018. The secondary outcomes were adherence to each component of the 1-h bundle therapy, resuscitation fluid type, vasopressor type, steroid administration, invasive arterial blood pressure monitoring, central-line insertion, intensive care unit (ICU) admission, length of stay in the emergency department, mechanical ventilator use, renal replacement therapy use, in-hospital mortality, and 28-day survival.

### Statistical analysis

Normally distributed and continuous variables were expressed as means and standard deviations and were compared using the Student’s *t*-test. Non-normally distributed and continuous variables were expressed as medians and interquartile ranges and compared using the Mann–Whitney *U* test. Categorical variables were expressed as numbers and percentages and compared using Fisher’s exact test or the chi-square test, as appropriate.

Propensity score matching was used to balance the variables between sexes. Standardized differences were used to evaluate the balance of the variables before and after propensity score matching. Variables with a standardized difference < 0.1 were considered balanced. Unbalanced variables were entered into a logistic regression model to calculate the sex-stratified propensity scores. The distribution and overlap of the propensity scores were evaluated before and after propensity score matching (Supplementary Fig. 1). Propensity score matching was performed using the 1:1 nearest neighbor-matching method with a caliper width of 0.2 without replacement. For the matched cohort, variables were compared using a statistical test for paired data.

Multivariable logistic regression analysis was performed in the matched and pre-matched cohorts to evaluate the independent association between sex and outcomes. The variables that were significant at a level of 0.1 in the univariable logistic regression analysis and the variables selected based on previous literature were entered into the multivariable logistic regression model. The variables selected based on previous literature were septic shock status^4,17^, infection focus^6,13,17^, APACHE II score^13,14^, and lactate^13,14^. A multivariate Cox proportional hazards model was used for analyzing 28-day survival.

Restricted cubic spline analysis was performed to evaluate the sex-stratified nonlinear relationship between age, APACHE II score, and adherence to 1-h bundle therapy. Four knots were used for the restricted cubic spline curve after adjusting for variables that were used in the multivariable logistic regression analysis. Subgroup analyses were performed according to septic shock status, age (≥ 65 or < 65), and SOFA score (≥ 8 or < 8).

As an exploratory analysis, the adjusted association between sex and adherence to bundle therapy was evaluated for each bundle therapy cutoff timepoint. Additionally, the association between adherence to bundle therapy according to each cutoff timepoint and 28-day survival was evaluated after adjustment.

Statistical significance was set at p < 0.05. R version 4.0.2 (R Foundation for Statistical Computing, Vienna, Austria) was used for statistical analysis.

### Ethics approval and consent to participate

This study was conducted in accordance with the principles of the Declaration of Helsinki. The Institutional Review Board of the Korea University Ansan Hospital approved this study (2023AS0109) and waived the requirement for informed consent because of the observational nature of the study.

## Results

**Figure 1:**
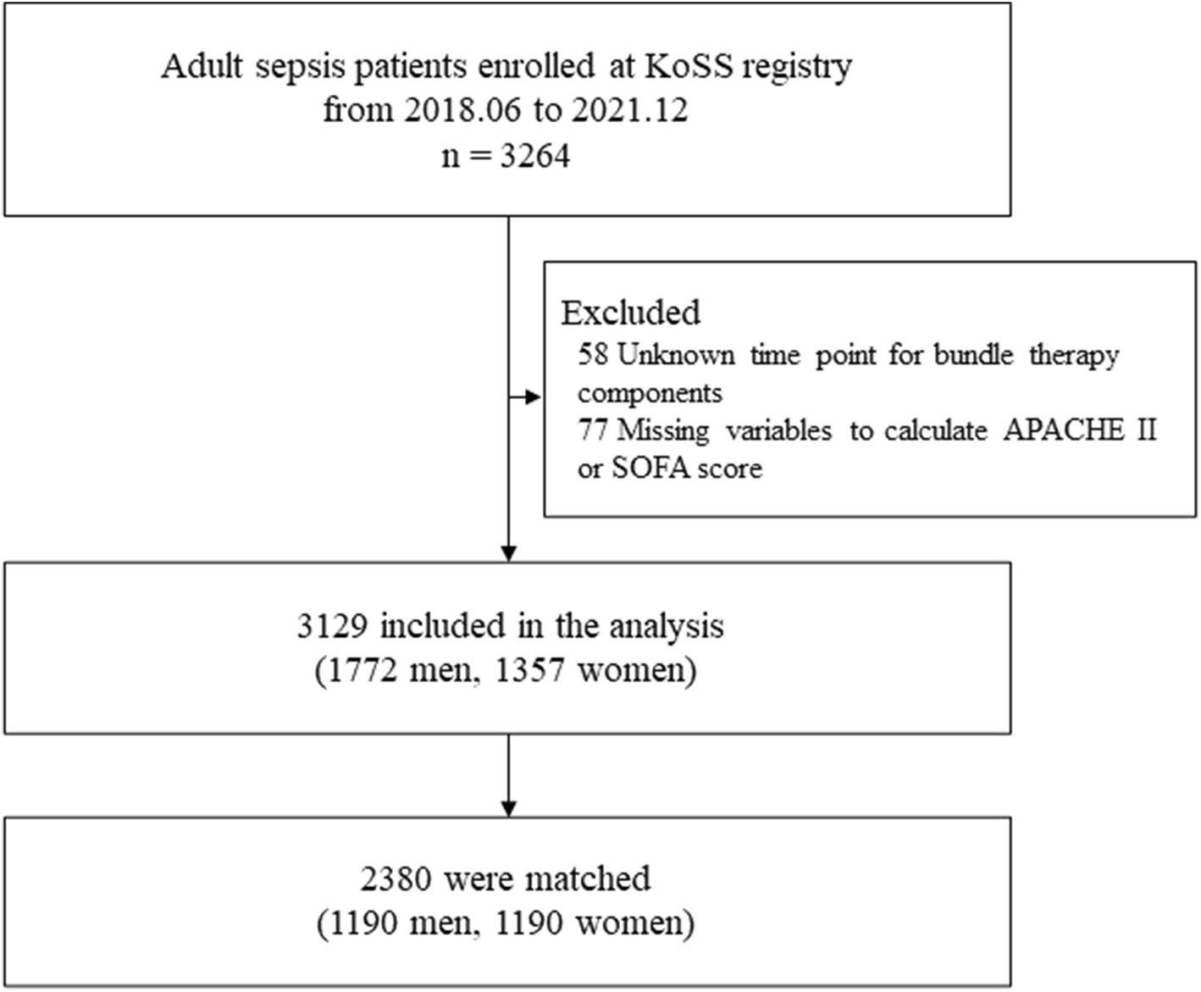
Flowchart of the study population. *KoSS* Korean Shock Society, *APACHE* Acute Physiologic Assessment and Chronic Health Evaluation, *SOFA* Sequential Organ Failure Assessment.

Between April 2018 and December 2021, 3,264 patients with sepsis were registered in the KoSS registry. Among them, 58 were excluded because of unknown timepoints for the bundle therapy component and 77 were excluded because of missing data on variables used to calculate the APACHE II or SOFA score. Finally, 3,129 patients were included in this study (Fig. 1).

In this study cohort, the mean age was 69.4 ± 12.6 years, 43.4% were women, the mean APACHE II score was 22 ± 9.2, the mean SOFA score was 8.9 ± 3.6, and 1,876 (60.0%) patients had septic shock. The in-hospital and the 28-day mortality rates were 26.8% and 25.2%, respectively.

### Before and after propensity score matching

In the analyses conducted before propensity score matching, the women were older and the men had higher APACHE II and SOFA scores and initial lactate levels. Respiratory infections, hepatobiliary infections, cardiac diseases, chronic lung diseases, and chronic liver diseases were more frequent in men than in women. Genitourinary infections and hypertension were more frequent in women than in men (Table 1). The 1-h bundle therapy was performed less frequently in women than in men (13.6% vs. 19.2%, *p* < 0.001). Among the bundle therapy components, broad-spectrum antibiotics were administered less frequently in women than in men (25.8% vs. 32.1%, *p* < 0.001), whereas adequate fluid resuscitation was performed more frequently in women than in men (96.7% vs. 93.9%, *p* = 0.001) (Table 2).

**Table 1:** Baseline characteristics of the pre-matched and matched cohorts.

	Before matching			After matching		
	Women (n = 1357)	Men (n = 1772)	Standardized difference	Women (n = 1190)	Men (n = 1190)	Standardized difference
Age (years)	72.0 [62.0–80.0]	70.0 [61.5–78.0]	0.112	71.0 [62.0–80.0]	70.0 [62.0–78.0]	0.037
Septic shock	791 (58.3%)	1085 (61.2%)	0.060	706 (59.3%)	721 (60.6%)	0.026
SOFA score	8 [6–11]	9 [7–12]	0.173	8 [6–11]	9 [7–11]	0.083
APACHE II score	21 [15–27]	21 [16–28]	0.102	21 [15–27]	21 [16–28]	0.050
Initial vital signs						
SBP (mmHg)	90.0 [75.0–112.0]	92.0 [78.0–114.0]	0.048	90.0 [75.0–113.0]	92.0 [78.0–115.0]	0.061
DBP (mmHg)	54.0 [46.0–67.0]	56.0 [48.0–68.0]	0.082	54.0 [46.0–67.0]	56.0 [48.0–68.0]	0.075
HR (1/min)	109.0 [92.0–126.0]	111.0 [95.0–128.0]	0.085	110.0 [92.0–127.0]	110.0 [94.0–127.0]	0.030
RR (1/min)	20.0 [18.0–24.0]	20.0 [18.0–24.0]	0.065	20.0 [18.0–24.0]	20.0 [18.0–24.0]	0.005
BT (℃)	37.7 [36.7–38.8]	37.6 [36.6–38.6]	0.102	37.7 [36.7–38.7]	37.7 [36.7–38.7]	0.006
Infection focus						
Respiratory	207 (15.3%)	529 (29.9%)	0.355	207 (17.4%)	217 (18.2%)	0.022
Genitourinary	384 (28.3%)	185 (10.4%)	0.464	217 (18.2%)	184 (15.5%)	0.074
Gastrointestinal	169 (12.5%)	201 (11.3%)	0.034	169 (14.2%)	156 (13.1%)	0.032
Hepatobiliary	204 (15.0%)	365 (20.6%)	0.146	204 (17.1%)	219 (18.4%)	0.033
Mixed	233 (17.2%)	280 (15.8%)	0.037	233 (19.6%)	236 (19.8%)	0.006
Others	160 (11.8%)	212 (12.0%)	0.005	160 (13.4%)	178 (15.0%)	0.043
Comorbidities						
HTN	631 (46.5%)	711 (40.1%)	0.129	549 (46.1%)	521 (43.8%)	0.047
DM	427 (31.5%)	597 (33.7%)	0.047	360 (30.3%)	409 (34.4%)	0.088
Cardiac disease	173 (12.7%)	305 (17.2%)	0.125	161 (13.5%)	161 (13.5%)	0.001
Chronic lung disease	57 (4.2%)	196 (11.1%)	0.261	57 (4.8%)	61 (5.1%)	0.015
Hematologic malignancy	103 (7.6%)	157 (8.9%)	0.046	98 (8.2%)	104 (8.7%)	0.018
Metastatic cancer	403 (29.7%)	605 (34.1%)	0.095	367 (30.8%)	416 (35.0%)	0.088
Chronic renal disease	123 (9.1%)	168 (9.5%)	0.014	113 (9.5%)	113 (9.5%)	0.001
Chronic liver disease	100 (7.4%)	204 (11.5%)	0.142	97 (8.2%)	97 (8.2%)	0.001
Laboratory data						
Lactate (initial) (mmol/L)	3.2 [1.9–5.5]	3.9 [2.2–5.9]	0.117	3.2 [2.0–5.6]	3.7 [2.1–5.7]	0.041
WBC (× 10^3^/μL)	10.0 [4.1–17.0]	10.0 [3.9–16.6]	0.035	9.7 [3.9–17.0]	10.0 [3.9–16.2]	0.041
CRP (mg/dL)	14.5 [6.2–25.0]	14.7 [6.4–24.6]	0.014	14.5 [6.2–25.3]	14.8 [6.5–24.6]	0.005

Data are expressed as median [interquartile range], mean ± standard deviation, or frequency (proportion), as appropriate.

*SOFA* Sequential Organ Failure Assessment, *APACHE* Acute Physiologic Assessment and Chronic Health Evaluation, *SBP* systolic blood pressure, *DBP* diastolic blood pressure, *HR* heart rate, *RR* respiratory rate, *BT* body temperature, *HTN* hypertension, *DM* diabetes mellitus, *WBC* white blood cell, *CRP* C-reactive protein.

**Table 2:** Outcomes of the pre-matched and matched cohorts.

	Before matching			After matching		
	Women (n = 1357)	Men (n = 1772)	*p*-value	Women (n = 1190)	Men (n = 1190)	*p*-value*
1-h bundle therapy	184 (13.6%)	341 (19.2%)	< 0.001	155 (13.0%)	228 (19.2%)	< 0.001
Initial lactate measurement	1158 (85.3%)	1535 (86.6%)	0.327	1011 (85.0%)	1013 (85.1%)	0.908
Blood culture before antibiotics	1126 (83.0%)	1494 (84.3%)	0.340	984 (82.7%)	1009 (84.8%)	0.159
Broad-spectrum antibiotics	350 (25.8%)	569 (32.1%)	< 0.001	302 (25.4%)	376 (31.6%)	< 0.001
Adequate fluid resuscitation	1312 (96.7%)	1664 (93.9%)	0.001	1152 (96.8%)	1131 (95.0%)	0.029
If hypotensive, vasopressors to achieve MAP ≥ 65 despite fluid resuscitation	1153 (85.0%)	1544 (87.1%)	0.091	1019 (85.6%)	1039 (87.3%)	0.239
Fluid						
Normal saline	939 (69.2%)	1152 (65.0%)	0.015	817 (68.7%)	784 (65.9%)	0.160
Balanced crystalloids	681 (50.2%)	891 (50.3%)	0.985	611 (51.3%)	614 (51.6%)	0.903
Albumin	192 (14.1%)	262 (14.8%)	0.653	173 (14.5%)	165 (13.9%)	0.639
Vasopressors						
Norepinephrine	1243 (91.6%)	1610 (90.9%)	0.509	1097 (92.2%)	1085 (91.2%)	0.414
Vasopressin	299 (22.0%)	432 (24.4%)	0.135	271 (22.8%)	272 (22.9%)	1.000
Steroid	363 (26.8%)	518 (29.2%)	0.136	324 (27.2%)	327 (27.5%)	0.887
Monitoring						
Invasive arterial blood pressure monitoring	1020 (75.2%)	1452 (81.9%)	< 0.001	904 (76.0%)	954 (80.2%)	0.013
Central-line insertion	830 (61.2%)	1024 (57.8%)	0.062	725 (60.9%)	683 (57.4%)	0.077
Clinical outcomes						
ICU admission	795 (58.6%)	1048 (59.1%)	0.782	700 (58.8%)	678 (57.0%)	0.353
ED length of stay (h)	9.8 [6.4–19.6]	9.8 [6.1–19.4]	0.566	9.8 [6.3–19.3]	10.4 [6.4–20.2]	0.094
Mechanical ventilator use	374 (27.6%)	615 (34.7%)	< 0.001	345 (29.0%)	369 (31.0%)	0.278
RRT use	200 (14.7%)	286 (16.1%)	0.306	185 (15.5%)	187 (15.7%)	0.909
In-hospital mortality	334 (24.6%)	504 (28.4%)	0.018	313 (26.3%)	311 (26.1%)	0.924
28-day survival	999 (73.6%)	1228 (69.3%)	0.009	861 (72.4%)	846 (71.1%)	0.477

Data were expressed as median [interquartile range] or frequency (proportion), as appropriate.

*MAP* mean arterial pressure, *ICU* intensive care unit, *ED* emergency department, *RRT* renal replacement therapy.

*Paired test (paired Mann–Whitney *U* test, McNemar’s test).

After propensity score matching, 1,190 women and 1,190 men were included in the analysis. All variables were well-balanced, with standardized differences of < 0.1 (Table 1). The 1-h bundle therapy was performed less frequently in women than in men (13.0% vs. 19.2%; *p* < 0.001). Among the bundle therapy components, compared to men, broad-spectrum antibiotics were administered less frequently (25.4% vs. 31.6%, *p* < 0.001) and adequate fluid resuscitation was performed more frequently in women (96.8% vs. 95.0%, *p* = 0.029) (Table 2).

### Multivariable logistic regression analysis of sex and 1-h bundle therapy

**Figure 2:**
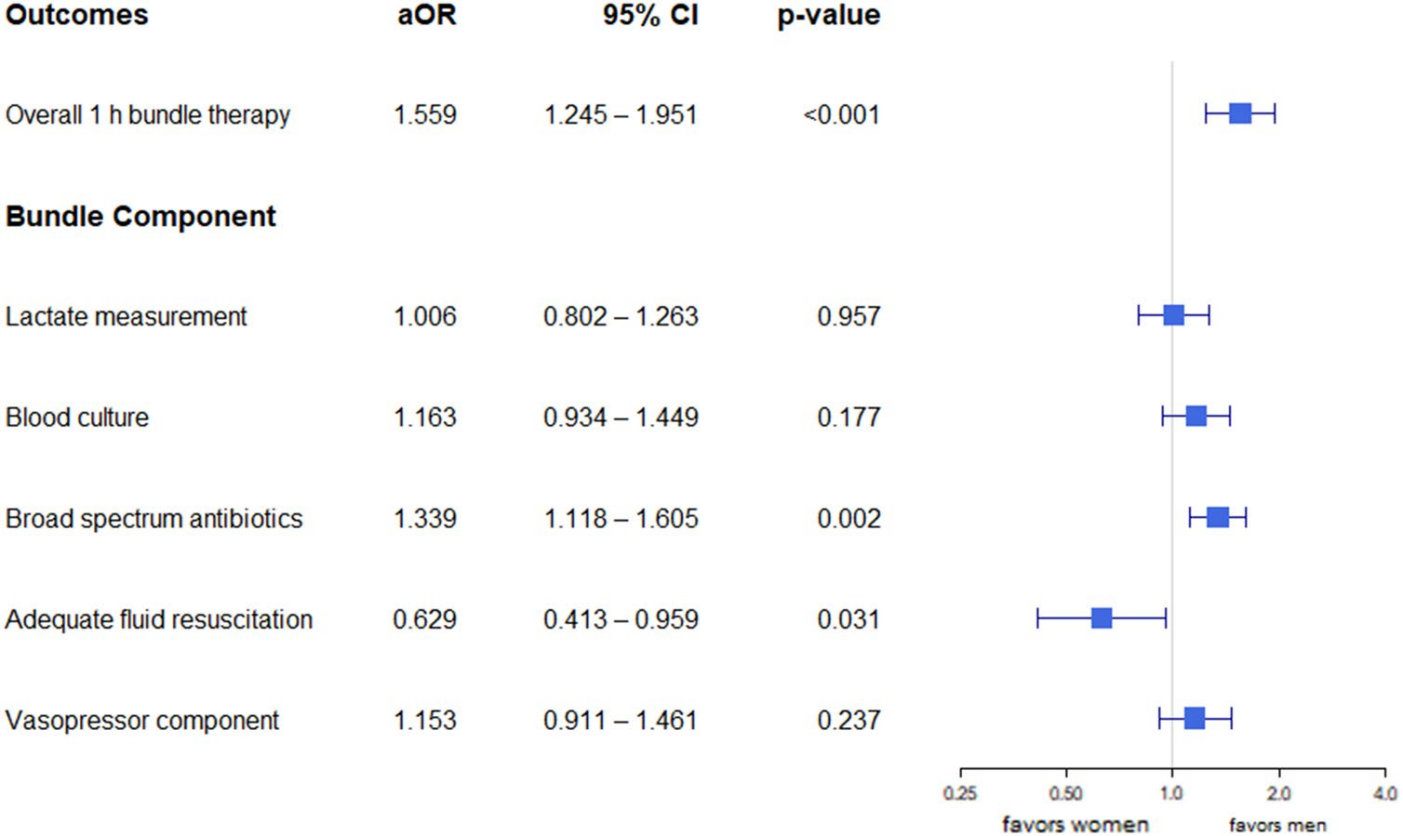
Multivariable logistic regression analysis of the matched cohort. Septic shock, infection focus (respiratory), APACHE II score, and lactate were adjusted in the multivariable model. aOR > 1 favors men. *APACHE* Acute Physiologic Assessment and Chronic Health Evaluation, *aOR* adjusted odds ratio, *CI* confidence interval.

After adjusting for the APACHE II score, lactate level, infection focus, and septic shock (Supplementary Table 1), 1-h bundle therapy was performed less frequently in women than in men [adjusted odds ratio (aOR) 1.559, 95% confidence interval (CI), 1.245–1.951; *p* < 0.001; Fig. 2]. Among the bundle therapy components, broad-spectrum antibiotics were administered less frequently in women than in men (aOR 1.339, 95% CI, 1.118–1.605; *p* = 0.002) whereas adequate fluid resuscitation was performed more frequently in women than in men (aOR 0.629, 95% CI, 0.413–0.959; *p* = 0.031). No sex difference was observed in any of the other components. Multivariable logistic regression analysis of the pre-matched cohort showed similar results (Supplementary Fig. 2).

### Secondary outcomes stratified by sex

Among the secondary outcomes, invasive arterial blood pressure monitoring was performed less frequently in women than in men, both before and after propensity score matching (75.2% vs. 81.9%, *p* < 0.001, and 76.0% vs. 80.2%, *p* = 0.013, respectively). Resuscitation fluid type, vasopressor type, steroid administration, central-line insertion, ICU admission, length of stay in the emergency department, mechanical ventilator use, renal replacement therapy use, in-hospital mortality, and 28-day survival were comparable for both the sexes (Table 2).

### Restricted cubic spline analysis

**Figure 3:**
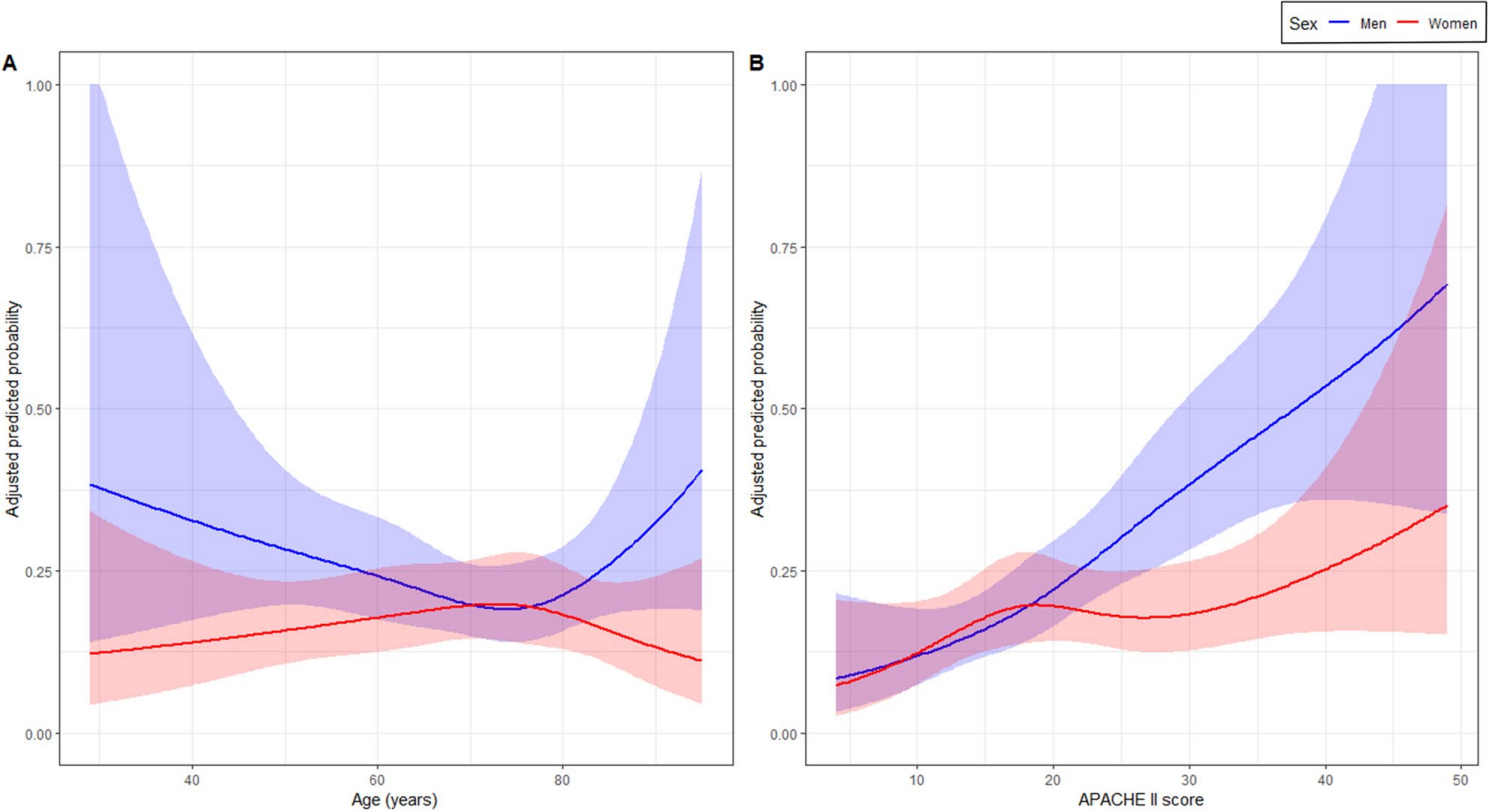
Sex-stratified adjusted predicted probability of adherence to the bundle therapy according to age (**A**) and APACHE II score (**B**). The shaded area shows the 95% confidence interval for the predicted probabilities point estimate. *APACHE* Acute Physiologic Assessment and Chronic Health Evaluation.

Based on the restricted cubic spline analysis of age, the adjusted predicted probability of adherence to the 1-h bundle therapy was higher for men than for women across all ages, except for those aged 70–80 years (Fig. 3A). Restricted cubic spline analysis of the APACHE II score showed that the adjusted predicted probability of adherence to 1-h bundle therapy and sex differences linearly increased with the APACHE II score. When the APACHE II score was > 20, the predicted probability of adherence to the 1-h bundle therapy was higher in men than in women (Fig. 3B).

### Subgroup analysis

**Figure 4:**
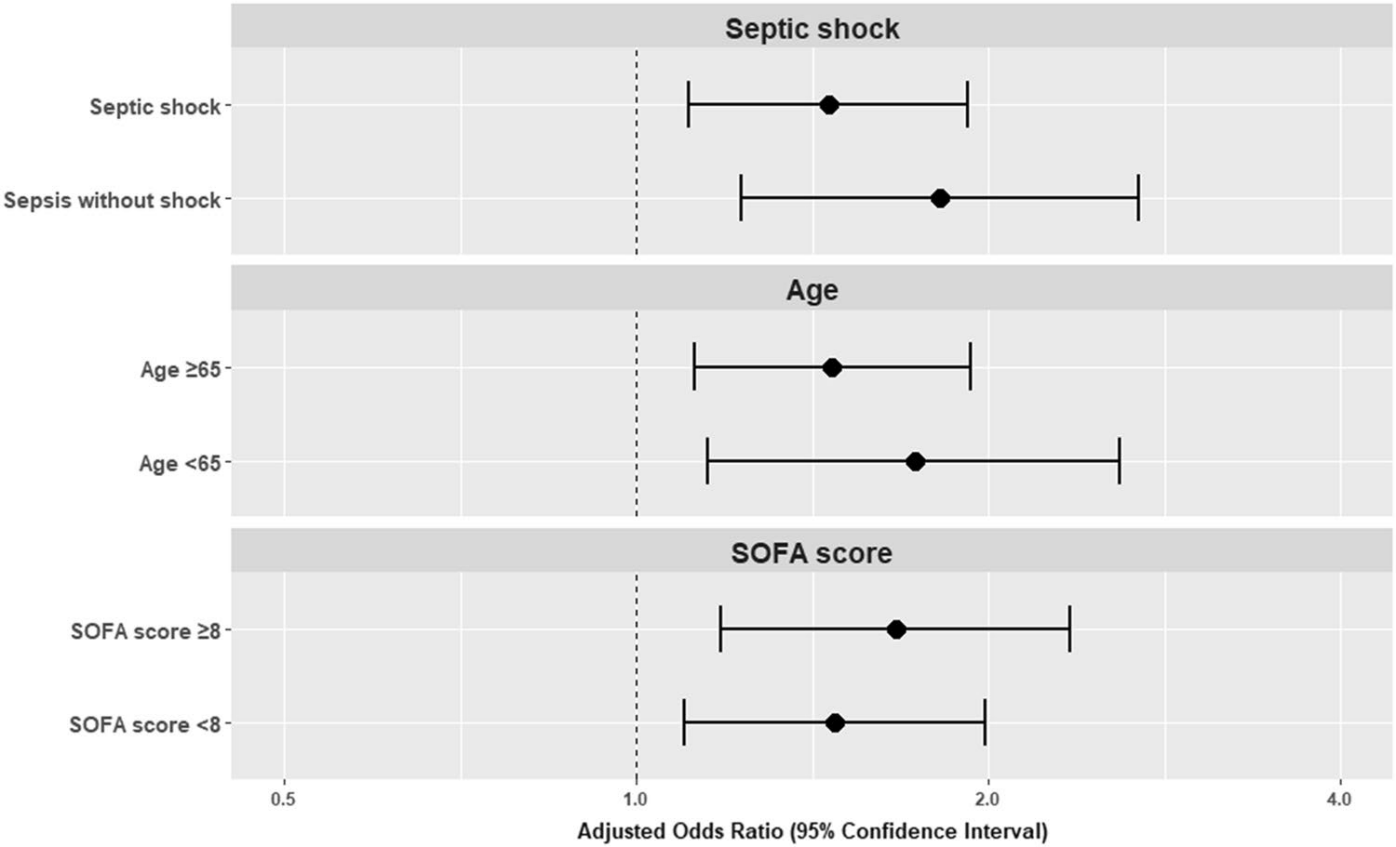
Subgroup analysis according to septic shock status, age, and SOFA score (matched cohort). aOR > 1 favors men. *SOFA* Sequential Organ Failure Assessment.

In the subgroup analyses, women were less likely to receive 1-h bundle therapy than men, regardless of septic shock status, age (≥ 65 or < 65), and the SOFA score (≥ 8 or < 8) (Fig. 4). Subgroup analyses of the pre-matched cohort showed similar results (Supplementary Fig. 3).

### Exploratory analysis

In the exploratory analysis, sex differences, wherein women were less likely to receive bundle therapy than men, persisted up to a cutoff timepoint of 98 min (Supplementary Fig. 4). Bundle therapy exhibited a protective effect on 28-day survival up to a cutoff of 3 h (Supplementary Fig. 5).

### Patient’s initial presentation in the emergency department

Women presented with altered mental status more frequently than men (*p* = 0.029). The initial presentation of febrile status, hypotension, tachypnea, and quick SOFA ≥ 2 was comparable for both the sexes (Supplementary Table 2).

## Discussion

In the present study, 1-h bundle therapy, according to the Surviving Sepsis Campaign of 2018, was performed less frequently in women than in men. Among the 1-h bundle components, broad-spectrum antibiotics were administered less frequently in women than in men, whereas adequate fluid resuscitation was performed more frequently in women than in men. Sex differences in adherence to the 1-h bundle therapy persisted in various subgroup analyses. Among the other in-hospital management methods, invasive arterial blood pressure monitoring was performed less frequently in women than in men; however, other in-hospital management methods and survival rates were similar for both the sexes.

The strength of our study is that we simultaneously evaluated the overall 1-h bundle therapy and each of its components, along with various in-hospital managements. Furthermore, our study included patients with sepsis, which was defined using the Sepsis-3 definition, and evaluated sex differences based on the recently proposed 1-h bundle therapy. Most previous studies either included patients with sepsis that was defined using previous definitions^6,8,12,13^ or evaluated the 3-h bundle therapy^7,12^. Additionally, we used a multicenter prospective registry and used propensity score matching for unbalanced covariables, including age, comorbidities, infection focus, and severity score. The results of the multivariable logistic regression analysis of the pre-matched and matched cohorts and the results of the subgroup analysis were similar, thus indicating the statistical robustness of the results.

In our study, there was low adherence to the overall 1-h bundle therapy in the emergency department. Although the rate of adherence to the administration of broad-spectrum antibiotics within 1 h was similar to that reported in a previous study^17^, this rate was the lowest among all components of the bundle therapy. The low adherence to the administration of broad-spectrum antibiotics within 1 h may be attributable to the initial presentation of patients with sepsis in the emergency department. Notably, more than 40% of patients with sepsis were not initially febrile, and approximately 24% of patients with sepsis initially had altered mental status. Therefore, emergency physicians should differentiate these patients from those with other acute and time-sensitive diseases. Performing imaging or laboratory tests to rule out other conditions could potentially delay the administration of broad-spectrum antibiotics in the emergency departments. The patient-to-medical staff ratio and emergency department overcrowding may be additional factors that contribute to the rate of adherence^14,18^. Prompt administration of broad-spectrum antibiotics to patients with suspected sepsis (even without ruling out other diseases or waiting for the results of other studies), increasing medical staff, and reducing overcrowding may improve adherence to the overall bundle therapy in emergency departments. Continuous efforts to increase adherence to the 1-h bundle therapy, especially focusing on the administration of broad-spectrum antibiotics, are required in emergency departments.

Our results showed sex differences in adherence to the 1-h bundle therapy. Among the bundle therapy components, the administration of broad-spectrum antibiotics was significantly less frequent among women. Similar to the results of our study, a previous study on severe sepsis and septic shock reported that women were less likely to receive 1-h bundle therapy and 1-h antibiotic administration^6^. Another study reported that antibiotic administration within 3 h was performed less frequently in women with severe sepsis and septic shock^12^. Previous studies on severe sepsis and septic shock have reported that the time to antibiotic administration was longer in women than in men^19,20^. Considering the initial presentation of patients with sepsis in our study, altered mental status was more frequent, even after propensity score matching for potential covariables, including severity score and infection focus. A more frequent initial presentation of altered mental status may result in more frequent brain imaging workups in women compared to men. This difference might have contributed to sex differences in bundle therapy adherence for up to 98 min, as observed in our exploratory analysis. Conversely, our study found that adequate fluid resuscitation was performed more frequently in women than in men. Due to this sex difference, more frequent instability of vital signs may occur in men, leading to more frequent invasive arterial blood pressure monitoring. Another explanation for the difference in adequate fluid resuscitation may be differences in body weight. Women tend to have a lower body weight than men; thus, supplementation of 30 mL/kg is easier to achieve in women, even if the same amount of fluid is infused in both sexes. This is supported by a previous study on septic shock which reported that the infused fluid per kilogram was higher in women, although the total amount of fluid was similar between sexes^21^. To improve adherence to overall 1-h bundle therapy and decrease sex differences, the improvement of broad-spectrum antibiotics administration within 1 h is required in women, whereas the improvement of adequate fluid resuscitation is required in men. However, further studies are needed to determine sex differences in each component of bundle therapy to generalize these results.

Mortality was similar between sexes in the present study. Sex differences in the mortality rates among patients with sepsis remain controversial. Although some studies have reported higher mortality rates among women^6,22,23^, others have reported higher mortality rates among men^7,24–26^. Furthermore, previous studies have reported no difference between the sexes^12,21,27^. Possible explanations for these results are described below. Although 1-h bundle therapy was performed less frequently in women, lactate measurement, blood culture before antibiotic administration, and application of vasopressors were well-performed components of bundle therapy in both sexes. Although adequate fluid resuscitation was more prevalent among women, the sex-difference effect of fluid might be compensated for by closer monitoring in men, such as invasive arterial blood pressure monitoring. Regarding sex differences in antibiotic use within 1 h, the protective effect of antibiotics for up to 3 h can lead to reduced sex differences in mortality rates. Although the administration of antibiotics within 1 h was associated with survival in patients with septic shock, this was not associated with survival in patients without septic shock^17^. Antibiotic administration within 3 h was associated with the survival of patients with sepsis^28^. Exploratory analysis revealed that the sex difference in bundle therapy reduced after 98 min, and adherence to bundle therapy for up to 3 h showed a survival benefit. As antibiotic administration is a significant contributing factor to adherence to bundle therapy, the protective effect of antibiotics within 3 h may be relevant to the results of the exploratory analysis. The protective effect of 3 h bundle may lead to similar mortality in both sexes. Furthermore, mortality was similar between sexes after balancing for SOFA score, APACHE II score, body temperature, infection focus, comorbidities, and lactate in propensity score matching analysis. The severity of the patients, which can be represented by SOFA score, APACHE II score, or lactate, might be a more contributing factor for mortality. Additionally, other unmeasured factors such as fluid balance or nutritional status during hospital admission might affect the mortality.

Additionally, the proportion of patients with septic shock and those with sepsis without shock can contribute to the sex differences in mortality rates. A previous study on septic shock found no sex-related differences in mortality^21^. The proportion of patients with septic shock was 17% in a previous study that reported sex-related differences in mortality due to sepsis^7^. This study also found no sex-related differences in mortality in a subgroup of patients with septic shock. Sex differences in mortality may not have been revealed in our study owing to the high proportion of patients with septic shock. Furthermore, uncollected variables in the ICU or after hospital admission may have contributed to the mortality in patients with sepsis. Therefore, further studies on sex-related differences in mortality rates are required.

This study has several limitations. First, the observational design confers the possibility that some covariables might have been missed and only associations could be identified. Second, although a multicenter prospective registry was used, the study was conducted in a single nation, which limits the generalizability of the results to other nations. Therefore, further multinational studies are warranted. Third, in-hospital managements can affect mortality. In addition, data on in-hospital management and patient status, such as fluid balance or nutritional status in the ICU or general ward, were not collected. Mortality results must be interpreted with caution and further studies are warranted to evaluate sex difference in mortality after adjustment of in-hospital managements. Fourth, as the KoSS registry is an emergency department–based prospective multicenter registry and only patients with acute-onset sepsis who visited the emergency department were included in this study, these results cannot be generalized to patients with delayed-onset sepsis or hospital-acquired sepsis. Fifth, the COVID-19 pandemic might affect clinical practice in the emergency department. We additionally evaluated the differences according to the COVID-19 pandemic in the study cohort. Overall adherence to 1-h bundle therapy was comparable between the COVID pandemic and the period before the COVID pandemic. Among the components of the 1-h bundle therapy, lactate measurement and obtaining blood cultures were less frequently performed, while the vasopressor component was more frequently performed during the COVID pandemic compared to the period before the COVID pandemic (Supplementary Table 3). The sex difference in 1-h bundle therapy existed in both periods. However, the sex difference was smaller during the COVID pandemic (before the COVID pandemic: men 20.8% vs. women 12.5%, p < 0.001; COVID pandemic: men 17.8% vs. women 13.5%, p = 0.043). The decreased sex difference during the COVID pandemic was attributed to decreased lactate measurement and obtaining blood cultures in men, rather than an improvement in adherence to bundle components in women. The COVID pandemic might have delayed lactate measurement and obtaining blood cultures. The sex difference in the overall adherence to 1-h bundle therapy remained similar to the main result after additional adjustment for the COVID pandemic (Supplementary Table 4); however, there may be unmeasured effects of the COVID pandemic on the sex difference in in-hospital management. Therefore, further studies are required.

## Conclusions

In patients with sepsis and septic shock, 1-h bundle therapy was performed less frequently in women than in men. Among the 1-h bundle component, broad-spectrum antibiotics were administered less frequently in women than in men, whereas adequate fluid resuscitation was performed more frequently in women than in men. Continuous efforts are needed to increase adherence to 1-h bundle therapy and decrease sex differences.

### Supplementary Information

Supplementary Information.

## Data Availability

The datasets used and analyzed in this study are available from the corresponding author on reasonable request.

## Abbreviations

aOR: Adjusted odds ratio
APACHE: Acute Physiologic Assessment and Chronic Health Evaluation
CI: Confidence interval
ICU: Intensive care unit
KoSS: Korean Shock Society
MAP: Mean arterial pressure
PSM: Propensity score matching
SOFA: Sequential Organ Failure Assessment
