# Supplementary material for: Sex differences in in-hospital management in patients with sepsis and septic shock: a prospective multicenter observational study
Source: Sci Rep. 2024 Feb 28;14:4900. doi: 10.1038/s41598-024-55421-x (PMC10901798; doi:10.1038/s41598-024-55421-x)
Supplement: Supplementary file 1 — Supplementary Information. [file 41598_2024_55421_MOESM1_ESM.docx]

**SUPPLEMENTARY FILES**

**Supplementary Figure 1. Distributions and overlapping of the propensity score before and after propensity score matching.**


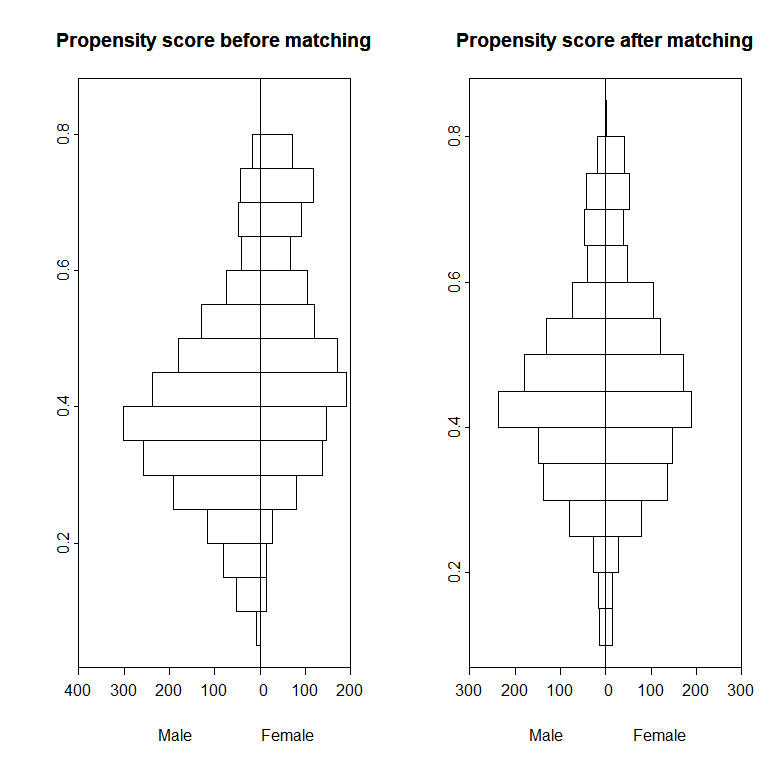


**Supplementary Figure 2. Multivariable logistic regression analysis of the pre-matched cohort.** Septic shock, infection focus (respiratory), APACHE II score, and lactate were adjusted in the multivariable model. aOR >1 favors men. APACHE, Acute Physiologic Assessment and Chronic Health Evaluation; aOR, adjusted odds ratio; CI, confidence interval.


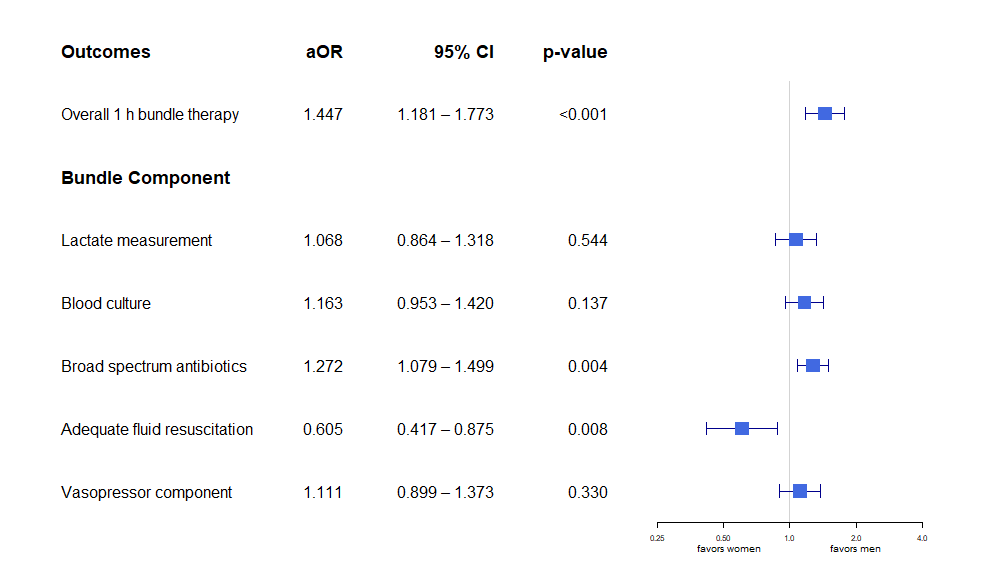


**Supplementary Figure 3. Subgroup analysis according to septic shock status, age, and SOFA score (pre-matched cohort).** aOR >1 favors men. SOFA, Sequential Organ Failure Assessment.


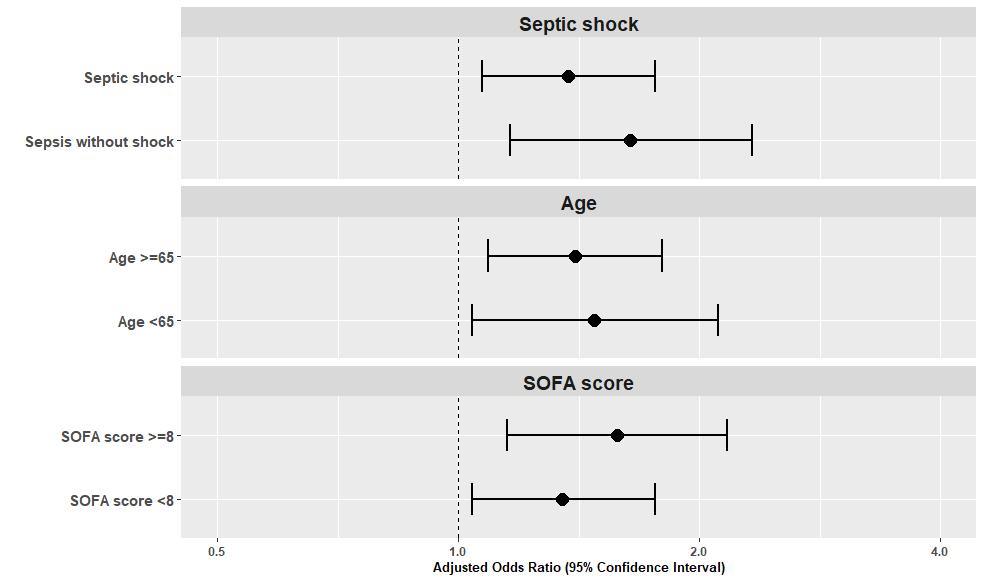


**Supplementary Figure 4. Adjusted sex difference in adherence to bundle therapy based on each cutoff timepoint.** aOR >1 favors men. The shaded area shows the 95% confidence interval. aOR, adjusted odds ratio.


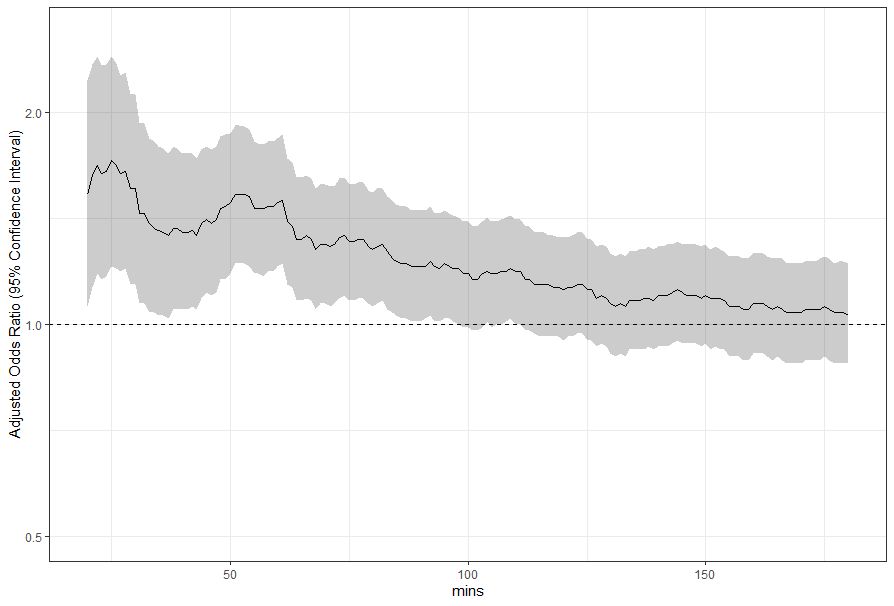


**Supplementary Figure 5. Adjusted protective effect of bundle therapy on the 28-day survival based on each cutoff timepoint.**


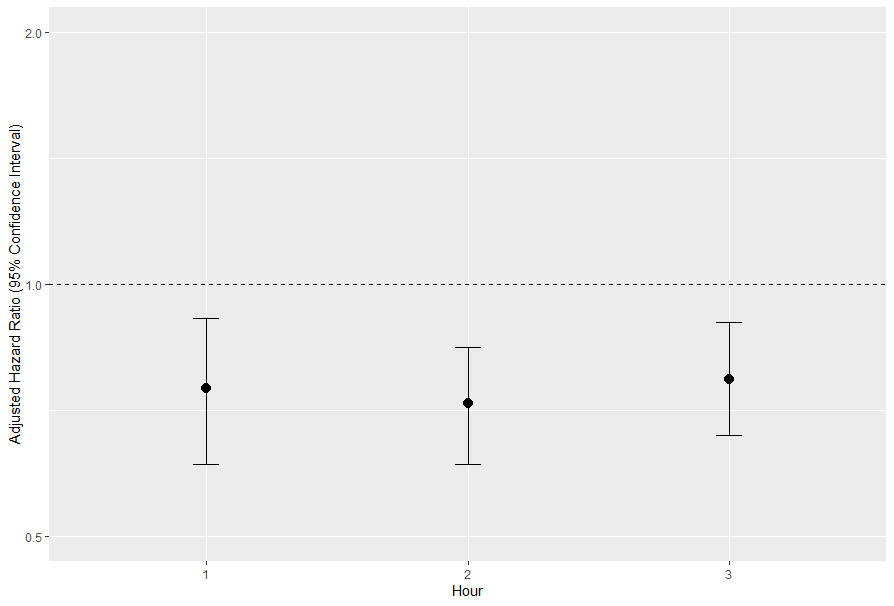


**Supplementary Table 1.** Univariable logistic regression analysis of bundle therapy (matched cohort)

| **Variables** | **OR** | **95% CI** | ***p*-value** |
| --- | --- | --- | --- |
| Age (years) | 1.001 | 0.993–1.010 | 0.786 |
| Sex (reference: women) | 1.583 | 1.269–1.979 | <0.001 |
| Septic shock | 1.422 | 1.131–1.795 | 0.003 |
| SOFA score | 1.093 | 1.059–1.136 | <0.001 |
| APACHE II score | 1.038 | 1.027–1.051 | <0.001 |
| **Initial vital signs** |  |  |  |
| SBP (mmHg) | 0.993 | 0.989–0.997 | 0.001 |
| DBP (mmHg) | 0.988 | 0.982–0.995 | <0.001 |
| HR (/min) | 1.005 | 1.001–1.009 | 0.025 |
| RR (/min) | 1.006 | 0.987–1.024 | 0.555 |
| BT (℃) | 1.022 | 0.945–1.106 | 0.585 |
| **Infection focus** |  |  |  |
| Respiratory | 1.269 | 0.962–1.659 | 0.087 |
| Genitourinary | 0.723 | 0.522–0.934 | 0.044 |
| Gastrointestinal | 0.890 | 0.634–1.224 | 0.485 |
| Hepatobiliary | 1.201 | 0.907–1.574 | 0.193 |
| Mixed | 1.091 | 0.829–1.424 | 0.526 |
| Others | 0.820 | 0.634–1.130 | 0.238 |
| **Comorbidities** |  |  |  |
| HTN | 1.062 | 0.852–1.323 | 0.590 |
| DM | 1.077 | 0.852–1.355 | 0.531 |
| Cardiac disease | 0.732 | 0.511–1.025 | 0.079 |
| Chronic lung disease | 1.206 | 0.732–1.1.907 | 0.440 |
| Hematologic malignancy | 1.662 | 1.165–2.333 | 0.004 |
| Metastatic cancer | 1.298 | 1.033–1.626 | 0.024 |
| Chronic renal disease | 0.781 | 0.514–1.148 | 0.227 |
| Chronic liver disease | 0.755 | 0.478–1.146 | 0.206 |
| **Laboratory data** |  |  |  |
| Lactate (initial) (mmol/L) | 1.026 | 0.993–1.059 | 0.120 |
| WBC (*10^3^/μL) | 1.000 | 1.000–1.000 | 0.232 |
| CRP (mg/dL) | 1.004 | 0.997–1.012 | 0.206 |

SOFA, Sequential Organ Failure Assessment; APACHE, Acute Physiologic Assessment and Chronic Health Evaluation; SBP, systolic blood pressure; DBP, diastolic blood pressure; HR, heart rate; RR, respiratory rate; BT, body temperature; HTN, hypertension; DM, diabetes mellitus; WBC, white blood cell; CRP, C-reactive protein.

**Supplementary Table 2.** Initial presentation of the study population in the emergency department

|  | **Before matching** | | | **After matching** | | |
| --- | --- | --- | --- | --- | --- | --- |
|  | Women  (n=1357) | Men  (n=1772) | *p*-value | Women  (n=1190) | Men  (n=1190) | *p*-value* |
| Febrile status | 734 (54.1%) | 894 (50.5%) | 0.047 | 629 (52.9%) | 635 (53.4%) | 0.806 |
| Hypotension | 646 (47.6%) | 804 (45.4%) | 0.228 | 567 (47.6%) | 531 (44.6%) | 0.149 |
| Dyspnea | 433 (31.9%) | 635 (35.8%) | 0.024 | 387 (32.5%) | 391 (32.9%) | 0.863 |
| Altered mental status | 361 (26.6%) | 376 (21.2%) | 0.001 | 311 (26.1%) | 266 (22.4%) | 0.029 |
| Initial qSOFA ≥2 | 522 (38.5%) | 667 (37.6%) | 0.664 | 452 (38.0%) | 449 (37.7%) | 0.900 |

*Paired test (paired Mann–Whitney *U* test, McNemar’s test).

qSOFA, quick Sequential Organ Failure Assessment.

**Supplementary Table 3.** Outcomes according to COVID pandemic period.

|  |  | Total |  |  | Women |  |  | Men |  |
| --- | --- | --- | --- | --- | --- | --- | --- | --- | --- |
|  | before COVID pandemic (n=1126) | COVID pandemic  (n=1254) | p-value | before COVID pandemic (n=582) | COVID pandemic  (n=608) | p-value | before COVID pandemic (n=544) | COVID pandemic  (n=646) | p-value |
| 1-h bundle therapy | 186 (16.5%) | 197 (15.7%) | 0.631 | 73 (12.5%) | 82 (13.5%) | 0.691 | 113 (20.8%) | 115 (17.8%) | 0.221 |
| Initial lactate measurement | 985 (87.5%) | 1039 (82.9%) | 0.002 | 504 (86.6%) | 507 (83.4%) | 0.142 | 481 (88.4%) | 532 (82.4%) | 0.004 |
| Blood culture before antibiotics | 978 (86.9%) | 1015 (80.9%) | <0.001 | 493 (84.7%) | 491 (80.8%) | 0.085 | 485 (89.2%) | 524 (81.1%) | <0.001 |
| Broad-spectrum antibiotics | 306 (27.2%) | 372 (29.7%) | 0.194 | 138 (23.7%) | 164 (27.0%) | 0.220 | 168 (30.9%) | 208 (32.2%) | 0.672 |
| Adequate fluid resuscitation | 1076 (95.6%) | 1207 (96.3%) | 0.454 | 560 (96.2%) | 592 (97.4%) | 0.336 | 516 (94.9%) | 615 (95.2%) | 0.887 |
| Vasopressor component | 956 (84.9%) | 1102 (87.9%) | 0.039 | 484 (83.2%) | 535 (88.0%) | 0.022 | 472 (86.8%) | 567 (87.8%) | 0.666 |

Data were expressed as frequency (proportion).

**Supplementary Table 4.** Multivariable logistic regression analysis of the matched cohort after additional adjustment of COVID-19 pandemic period.

|  | aOR | 95% CI | p-value |
| --- | --- | --- | --- |
| 1-h bundle therapy | 1.564 | 1.249–1.958 | <0.001 |
| Initial lactate measurement | 1.018 | 0.811–1.278 | 0.878 |
| Blood culture before antibiotics | 1.184 | 0.950–1.486 | 0.134 |
| Broad-spectrum antibiotics | 1.336 | 1.115–1.601 | 0.002 |
| Adequate fluid resuscitation | 0.621 | 0.407–0.948 | 0.027 |
| Vasopressor component | 1.143 | 0.902–1.449 | 0.267 |

Septic shock, infection focus (respiratory), APACHE II score, lactate, and COVID-19 pandemic period were adjusted in the multivariable model. aOR >1 favors men. APACHE, Acute Physiologic Assessment and Chronic Health Evaluation; aOR, adjusted odds ratio; CI, confidence interval.
